# Corrigendum to “Fatty Acid Profiles of Stipe and Blade from the Norwegian Brown Macroalgae Laminaria Hyperborea with Special Reference to Acyl Glycerides, Polar Lipids, and Free Fatty Acids”

**DOI:** 10.1155/2021/1207139

**Published:** 2021-01-16

**Authors:** Lena Foseid, Hanne Devle, Yngve Stenstrøm, Carl Fredrik Naess-Andresen, Dag Ekeberg

**Affiliations:** Faculty of Chemistry, Biotechnology and Food Science, Norwegian University of Life Sciences, P.O. Box 5003, 1432 Ås, Norway

In the article titled “Fatty Acid Profiles of Stipe and Blade from the Norwegian Brown Macroalgae Laminaria Hyperborea with Special Reference to Acyl Glycerides, Polar Lipids, and Free Fatty Acids” [1], the authors identified a systematic error in the calculations of the fatty acid contents. This error impacts the values in the text as well as both the tables and the figures. Therefore, while the conclusions of the article are not affected, the reported results are slightly altered. The authors apologize for this error, and the corrected version of the article is shown below:

 

 

A thorough analysis of the fatty acid profiles of stipe and blade from the kelp species *Laminaria hyperborea* is presented. Lipid extracts were fractionated into neutral lipids, free fatty acids, and polar lipids, prior to derivatization and GC-MS analysis. A total of 42 fatty acids were identified and quantified, including the *n*-3 fatty acids, *α*-linolenic acid, stearidonic acid, and eicosapentaenoic acid. The fatty acid amounts are higher in blade than in stipe (6.10 mg/g dry weight and 2.00 mg/g dry weight, respectively). The highest amounts of *n*-3 fatty acids are found within the neutral lipid fractions with 563.4 *μ*g/g dry weight and 97.4 *μ*g/g dry weight for blade and stipe, respectively. The amounts of polyunsaturated fatty acids are 3.3 times higher in blade than stipe. The blade had the highest PUFA/SFA ratio compared to stipe (1.18 versus 1.00) and the lowest *n*-6/*n*-3 ratio (0.8 versus 3.6). This study highlights the compositional differences between the lipid fractions of stipe and blade from *L. hyperborea*. The amount of polyunsaturated fatty acids compared to saturated and monounsaturated fatty acids is known to influence human health. In the pharmaceutical, food, and feed industries, this can be of importance for production of different health products.

## 1. Introduction

The increase in the world population and lack of sufficient food begs for new sources of food and feed. As much as 60% of the world food energy intake is provided by the cereals wheat, rice, and corn [2], these cereals, while high in metabolizable energy and carbohydrates, have small amounts of important nutrients such as proteins, minerals, vitamins (especially A and C), and fatty acids, especially long chained polyunsaturated *n*-3 fatty acids [3–5]. A promising supplement for food and feed is a better utilization of marine resources. World production and harvesting of micro- and macroalgae have doubled from 2004 to 2014 [6]. Still, 97% of the production and harvesting is found in Asia [6]; thus, there is a large potential for expansion in other parts of the world. Macroalgae is a diverse group of marine plants, informally divided into three groups: rhodophyta (red algae), chlorophyta (green algae), and phaeophyta (brown algae). Use of seaweed as feed, food, and fertilizers in times of food shortage was common in northern Europe from around the 10^th^ century and up until about middle of the 18^th^ century [7]. At present time in Scandinavia and other Western countries, the utilization of seaweeds is limited to industrial products such as alginate, agar, carrageenan and thickeners, and only scarcely used in food and feed industries. Biomarine processing industries have great potential in coastal regions. Norway is particularly privileged due to the long coastline combined with the presence of nutritious ocean currents (North Atlantic Drift and Norwegian Coastal Current), which ensures a good climate for growth of marine flora and fauna.

Seaweed has for many years been thought to have a positive effect on human health, and consumption of these marine plants has been linked to a lower incidence of cancer, hyperlipidemia, and coronary heart disease [8]. They are also reported to possess antimicrobial, antiviral, anti-inflammatory, and immunotropic properties [9]. Many of the reported medicinal effects of marine algae have not been confirmed, but Brown et al. and Stein & Borden have published comprehensive reviews on this topic [8, 10]. Lipid profiles and compositions can be of importance for human health and for commercial application [11–13]. Eukaryotic algae contain a diverse composition of acyl lipids and their fatty acids; albeit, the number of algae which have been comprehensively studied are relatively few [12]. Lipids in macroalgae can be divided into neutral lipids that include monoglycerides, diglycerides, triglycerides, sterols, and polar lipids that include glycolipids, phospholipids, and betaine lipids [11, 12, 14]. An important nutritional benefit of marine macroalgae is attributed to the high level of polyunsaturated fatty acids (PUFAs), especially *n*-3 and *n*-6 fatty acids [15]. A diet with a low *n*-6/*n*-3 ratio is reported to have suppressive effects on cardiovascular diseases, cancer, and inflammatory and autoimmune diseases [16]. With a global decline in fish stocks, seaweed may be a good option for an alternative and sustainable source of *n*-3 PUFAs [8]. Previous studies regarding fatty acids in brown macroalgae have had a nutritional or pharmaceutical focus. The fatty acid content has only been determined as one among several parameters, resulting in limited fatty acid profiles [17–21]. More extensive FA profiling has been done on certain species of brown macroalgae [9, 22–24]. Little is however known about fatty acid compositions of individual lipid classes of marine macrophytes in general [9]. The brown macroalgae *Laminaria hyperborea*, a common species of kelp found in the northern Atlantic, has only previously been characterized with regard to fatty acids in three studies [7, 13, 25], all presenting limited profiles identifying no more than nine fatty acids. The within-plant fatty acid distribution for this species has previously only been reported by Schmid and Stengel [25] with *Laminaria hyperborea* harvested on the west coast of Ireland. Variations in distribution of fatty acids between different parts of the seaweed depend on the morphology of the species as well as its physiological and ecological circumstances [26].

Before considering the marine algae as a food source, it is important to assess its nutritional value. In this context, the aim of this study has been to provide a thorough analysis of the fatty acid profiles in stipe and blade from the macroalgae *L. hyperborea*. In this study, the lipids were fractionated into free fatty acids (FFAs), acyl glycerides (neutral lipids, NLs), and polar lipids (PLs) by using solid phase extraction. The fatty acids from each class were identified and quantified by GC-MS.

## 2. Material and Methods

Chloroform and hexane were of Chromasolv® quality, heptane, diethyl ether, methanol, and NaCl puriss pa. quality, all from Sigma-Aldrich (St. Louis, MO, USA). The acetic acid was from Honeywell Riedel-de Haen (Seelze, Germany).

### 2.1. Standards

A fatty acid methyl ester (FAME) mix with 37 components (Food Industry Fame MIX, Restek, Bellefonte, PA, USA) was used for identification of the FAMEs. A 21 component FAME mix (Qualmix PUFA Fish M, Methyl Esters (Menhaden Oil), Larodan AB, Solna, Sweden) was used for identification of all-*cis*-6,9,12,15-octadecatetraenoic acid methyl ester, all-*cis*-8,11,14,17-eicosatetraenoic acid methyl ester, and all-*cis*-7,10,13,16,19-docosapentaenoic acid methyl ester. In addition, the following individual FAME standards were used: nonanoic acid methyl ester (Sigma-Aldrich, St. Louis, MO, USA), 13-methyltetradecanoic acid methyl ester, *trans*-9-tetradecenoic acid methyl ester, *cis*-9-heptadecenoic acid methyl ester, *cis*-13-octadecenoic acid methyl ester, *cis*-9-eicosenoic acid methyl ester, and hexacosanoic acid methyl ester (all from Larodan AB, Solna, Sweden). Three internal standards were used (10 mg/mL, dissolved in CHCl_3_), one for each lipid fraction, 1,2-dinonadecanoyl-sn-Glycero-3-phosphatidylcholine for the PL fractions, nonadecanoic acid for the FFA fractions, and trinonadecanoin for the NL fractions (all from Larodan AB, Solna, Sweden). Nonadecanoic acid methyl ester (Larodan AB, Solna, Sweden) was added to the 37 components FAME mix for retention time identification, since C19:0 was used as internal standard in the samples.

### 2.2. Pretreatment of L. Hyperborea

*L. hyperborea* was provided and identified by FMC BioPolymer AS. It was harvested off the west coast of Norway, outside Sør-Trøndelag County Municipality in October 2015. On board, the trawler *L. hyperborea* was rinsed, crude-cut, and preserved with formalin manually. The holdfast was discarded. Once off, the trawler stipe and blade were vacuum packed separately. When received at the university, stipe and blade were rinsed with water to eliminate contaminants, frozen with liquid N_2_ (99.999%, AGA, The Linde Group, Munich, Germany), and freeze-dried (Alpha 2-4 LD plus, Martin Christ Gefriertrocknungsanlagen GmbH, Osterode am Harz, Germany). The freeze-dried material was crushed in a QMM Micro–Mixer and pulverized in a Laboratory Mixer 3100 (Danfoss) by G. A. Lund at Pharmatech AS, Fredrikstad, Norway. The water content of fresh seaweed was measured according to ISO 11465 : 1993/Cor1 : 1994.

### 2.3. Lipid Extraction

Four sample replicates of both stipe and blade were used, and they were all treated separately during the sample preparation stages. The lipids were extracted with a modified Folch's method [27]. In brief, 5–10 g alga powder was extracted in a separatory funnel with 10 times its volume CHCl_3_:MeOH (2/1), and 50 *μ*L of each internal standard was added with a Hamilton® syringe. To induce phase separation, 0.9% NaCl was added after mixing (0.2 times the volume of CHCl_3_:MeOH). After approximately 20 min, the organic phase was transferred to a test tube (Duran® 20 x 150 mm, Mainz, Germany). The polar phase was reextracted with CHCl_3_, 30–60 mL depending on the amount of alga powder. The organic phases of each sample were combined and evaporated with a vacuum evaporator (Q-101, Buchi Labortechnik AG, Flawil, Switzerland) at 40°C, redissolved in 1.00 mL chloroform, and transferred to vials for SPE.

A liquid-handling robot (Gilson, GX-271, ASPEC, Middleton, WI, USA) was used to carry out the SPE procedure. The method used was based on previous work by Pinkart et al. 1998 and Ruiz et al. 2004 [28, 29]. Aminopropyl-modified silica phase SPE columns, 500 mg, 3 mL, (Chromabond, Macherey-Nagel, Düren, Germany) were conditioned with 7.5 mL hexane before 500 *μ*L of sample was applied. The NLs (mono-, di-, and triglycerides) were eluted with 5 mL chloroform, then the FFAs with 5 mL diethyl ether : acetic acid (98 : 2 v/v), and lastly, the PLs with 5 mL methanol. The possibility of cross-contaminations between any of the three classes was checked by performing tests with standards for each lipid class. The recovery was 90% or higher. The eluates were transferred to culture tubes (Duran 12 × 100 mm, Mainz, Germany) and evaporated under N_2_ (g) at 40°C.

### 2.4. Formation of FAMEs

For formation of FAMEs, the NL and PL fractions were redissolved in 2 mL of heptane, before addition of 1.5 mL of 3.3 mg/mL sodium methoxide. The sodium methoxide solution was made by dissolving metallic sodium (purum, Merck, Darmstadt, Germany) in methanol to a concentration of 3.3 mg/mL. The culture tubes were then shaken horizontally for 30 min at 350 rpm (Biosan Ltd, PSU-10i, Riga, Latvia) and left to settle vertically for 10 min before the heptane phases were transferred to vials for storage at -20°C prior to GC-MS analysis. The FFA fractions were redissolved in 1 mL BF_3_-MeOH (14%, Sigma-Aldrich, St.Louis, MO, USA). The samples were heated for 5 min at 70°C in a water bath. After heating, 1 mL heptane was added to each sample tube before mixing on a vortex mixer. The heptane phases were transferred to vials and stored at -20°C prior to analysis by GC-MS.

### 2.5. Analysis of FAMEs by GC-MS

The analysis of the FAMEs was based on a previously published method [30]. Shortly, the analysis were carried out on an Agilent 6890 Series Gas Chromatograph (GC; Agilent Technology, Wilmington, DE, USA) in combination with an Autospec Ultima mass spectrometer (MS; Micromass Ltd., Manchester, England) using an EI ion source. The GC was equipped with a CTC PAL Autosampler (CTC Analytics, AG, Zwingen, Switzerland). Separation was carried out on a 60 m Restek column (Rtx®-2330) with 0.25 mm I.D. and a 0.2 *μ*m film thickness of fused silica 90% biscyanopropyl/10% phenylcyanopropyl polysiloxane stationary phase (Restek Corporation, Bellefonte, PA, USA). Carrier gas helium (99.99990%, from Yara, Rjukan, Norway) was used at 1 mL/min constant flow. The EI ion source was used in positive mode, producing 70 eV electrons at 250°C. The MS was scanned in the range 40–600 m*/z* with 0.3 s scan time, 0.2 s inter scan delay, and 0.5 s cycle time. The transfer line temperature was set at 270°C. The resolution was 1000.

A split ratio of 1/10 was used with injections of 1.0 *μ*L sample. Two injections parallels were used for each sample replicate. Identification of fatty acids was performed by comparing retention times with standards as well as MS library searches. MassLynx version 4.0 (Waters, Milford, MA, USA) and NIST 2014 Mass Spectral Library (Gaithersburg, MD, USA) were used. Relative response factors previously determined by Devle et al. [30] were employed for quantitative determination. The resulting amounts are given in *μ*g/g dry weight (DW). The GC oven had a start temperature of 65°C, held for 3 min, the temperature was then raised to 150°C (40°C/min), held for 13 min, before being increased to 151°C (2°C/min), and held for 20 min, with a slow increase to 230°C (2°C/min) and held for 10 min, before a final increase to 240°C (50°C/min), and the end temperature was held for 3.7 min.

## 3. Results and Discussion

We have identified and quantified 42 different fatty acids in *L. hyperborea*, as shown in Table 1. This is a significantly higher number than previously reported by others for this species [7, 13, 25]. Seaweeds usually contain a lipid level of <1–5% [20, 22, 31]. The portions of the total lipids that contain molecules with fatty acids depend significantly on species, geographical location, and seasonal changes [13, 19, 25, 32, 33]. In our study, the total FA (TFA) content relative to dry weight in blade and stipe was 0.61% and 0.20%, respectively. This 3 : 1 ratio between blade and stipe is consistent with Schmid and Stengel [25] findings of within-plant variations for the same species, even though they had twice the TFA content (0.5% and 1.5% in stipe and blade, respectively). The water content was found to be 83.3% ± 0.5 and 85.6% ± 0.8 in blade and stipe, respectively. The fatty acid profile was determined for the NL, FFA, and PL fractions in stipe and blade separately. For the individual lipid fractions, the %TFA was the highest in NLs with 49.6% and 59.1% in stipe and blade, respectively. The PL fraction for the stipe consisted of 40.5% TFA versus 25.6% TFA in blades. The %TFA for the FFAs ranged from 9.8% in stipe to 15.4% in blade. While up to 41 different fatty acids were detected within a lipid fraction, the same 10 fatty acids predominated in all fractions in both stipe and blade. In this group of 10, three FAs were saturated (SFA, C14:0, C16:0 and C18:0), two were monounsaturated (MUFA, C16:1 *cis*-9, and C18:1 *cis*-9), and the remaining five were polyunsaturated (PUFA, C18:2 *cis*-9,12, C18:3 *cis*-9,12,15, C18:4 *cis*-6,9,12,15, C20:4 *cis*-5,8,11,14, and C20:5 *cis*-5,8,11,14,17).

The predominating fatty acids constitute more than 90% of the total fatty acid content in all the fractions, as shown in Figure 1. They are found in amounts varying from 0.61 to 1090 *μ*g/g DW (Table 1). A fatty acid was classified as predominating if it was above 2% of the total fatty acid content in at least one of the lipid fractions in either stipe or blade. All these ten fatty acids corresponded to those identified by others [7, 13, 25]. Schmid and Stengel [25] identified C18 : 3*n*-6, at 1.2% in stipe and 5.5% in blade, which differs from our results where C18 :3*n*-6 is not above 2% in any of the blade lipid fractions. This could be due to geographical and/or seasonal variations. The same FA was not detected at all by van Ginneken et al. [13] and also not reported by Maehre et al. [7], who both studied *L. hyperborea* as one of several macroalgal species. Only a maximum of two *trans*-fatty acids, C14:1 *trans*-9 and C16:2 *cis or trans*-7,10, were identified in the samples, both in relatively low amounts (≤1.63 *μ*g/g DW). The predominating fatty acids are important dietary *n*-3 fatty acids such as *α*-linolenic acid (ALA, C18:3*n*-3), stearidonic acid (SDA, C18:4*n*-3), and eicosapentaenoic acid (EPA, C20:5*n*-3) as well as two *n*-6 fatty acids, linoleic acid (LA, C18:2*n*-6), and arachidonic acid (AA, C20:4*n*-6). How favorable *L. hyperborea* is for the human diet (and thus also in animal feed) and depends on the several factors, for example, content of essential FAs (LA and ALA), other important nutritional FAs (SDA, AA, EPA), and the ratios between PUFA/SFA and *n*-6/*n*-3 fatty acids.

It is known that brown seaweeds grown in temperate or subarctic areas can accumulate *n*-3 and *n*-6 PUFAs [34]. In both stipe and blade, the lowest amounts of *n*-3 and *n*-6 fatty acids are found in the FFA fraction, while the highest amounts are found in the NL fraction. The highest amounts of the essential FAs, LA, and ALA were found in the NL fraction of blade (165.5 *μ*g/g DW and 74.8 *μ*g/g DW, respectively). For stipe, the highest amount of LA and ALA were also found in the NL fraction (40.3 and 6.5 *μ*g/mL DW, respectively). There was a lower proportion of the *n*-6 FA arachidonic acid in blade versus stipe (11.1% and 20.9%, respectively). This corresponds to what was reported by Schmid and Stengel [25].

The World Health Organization (WHO) recommends a daily intake of 0.25 g EPA+C22 : 6 *n*-3 (DHA) as part of a healthy diet [35]. Even though seaweed can have high levels of EPA, the fatty acid DHA is generally absent or only found in small amounts in different phaeophytes [19]. The highest amount of DHA in our study was found in the blade FFA fraction (1.84 *μ*g/g DW). With a total content of EPA + DHA in blade at 734 *μ*g/g DW, achieving the recommended amount by consumption of seaweed alone is highly unlikely, as the daily intake would have to be approximately 300 g dried seaweed (or 1500 g fresh seaweed). Although *n*-3 and *n*-6 PUFAs are usually easily oxidized, studies have shown that these PUFAs have exhibited high oxidative stability in seaweed lipids in dried seaweed products [36]. The reason for this might be due to a protective effect of galactosyl and sulfoquinovosyl moieties on PUFAs bonded to glycoglycerolipids (the main membrane lipids) [34].

Stipe and blade differ not only in the content of the individual FAs but also in the amounts of SFAs, MUFAs, and PUFAs (Table 2). The TFA in stipe was distributed evenly between the SFA, MUFA, and PUFA with 33.3%, 33.5%, and 33.3%, respectively. The blades, however, had a higher distribution of PUFAs with 36.3% of TFA and a slightly lower distribution of SFA and MUFA with 30.8% and 33.0%, respectively. Schmid and Stengel [25] also reported a higher distribution of PUFAs in blade versus stipe with 52.0% and 32.2%, respectively. The FA distribution differs significantly with geographical and seasonal variations, most likely due to nutrition, light conditions, and other biological factors. van Ginneken et al. [13] found a PUFA distribution of 53% of TFA in *L. hyperborea* (fronds) harvested in France with 25% SFA and 22% MUFA. Maehre et al. [7] who investigated the same species (whole plant) but harvested from the Norwegian coast reported 34.2% PUFA, 33.7% SFA, and 26.5% MUFA. These results are very similar to our findings, even though there was a significant distance in time of harvest (May/June 2010 versus October 2015). The total amount of PUFAs in blade is 3.3 times higher than in stipe, and the total amounts of SFAs and MUFAs are 2.8 and 3.0 times higher, respectively. PUFAs are preferred over SFAs from a dietary perspective, and replacing SFAs with PUFAs in the diet decrease the risk of coronary heart disease [35]. The NL fraction in stipe and the PL fraction in blade have the highest PUFA/SFA ratio of 2.18 and 1.45, respectively. When combining the lipid fractions, the blade FAs had a higher PUFA/SFA ratio compared to the stipe FAs (1.18 versus 1.00).

*L. hyperborea* has a *n*-6/*n*-3 ratio of 0.8/1 in blade and 3.6/1 in stipe. The ratios vary between the lipid fractions, as seen in Table 2, but are higher in stipe than in blade. In Western diet, the *n*-6/*n*-3 ratio is 15-20/1, and this is significantly higher than ~1, which was normal during human evolution [16, 37]. However, for health benefits, lowering this ratio is considered to be beneficial and associated with prevention of inflammatory, cardiovascular, and neural disorders [13]. *n*-6/*n*-3 ratios of 2-5/1 are reported to have suppressive effects on cardiovascular, inflammatory, and autoimmune diseases [16, 38, 39]. Since there is a significant difference between the *n*-6/*n*-3 ratios in stipe and blade, using only blade could be considered if a very low *n*-6/*n*-3 ratio is desired. Though, in this context, it should also be mentioned that FAO in their 2010 report [35] do not consider this ratio to be important and gives no specific recommendations of such.

While the same fatty acids predominate, the amounts in blade are consistently higher than those in stipe, as seen in Figure 2. This is consistent with results found by Schmid and Stengel [25]. At minimum, the amounts in blade are 1.1 times higher than in stipe for myristic acid (C14 : 0) in the PL fraction, and at maximum, 69.3 times higher than in stipe for SDA in the FFA fraction. For the FFA and PL fractions, the largest differences are found in the fatty acids ALA, SDA, and EPA. In the NL fraction, the largest difference between stipe and blade amounts is found in stearic acid (C18:0), while blade and stipe amounts are almost equal in the FFA and PL fractions for the same fatty acid.

## 4. Conclusions

A total of 42 different fatty acids are identified and quantified in the stipe and blade of *L. hyperborea*, with maximum two fatty acids having *trans*-configuration. Some fatty acids are found either in stipe or blade, while others are only present in certain lipid fractions (NL, FFA, PL) within stipe and blade. Among the predominating fatty acids are the *n*-3 fatty acids ALA (8.8 and 113 *μ*g/g DW), SDA (13.1 and 297 *μ*g/g DW), and EPA (112 and 731 *μ*g/g DW), as well as two *n*-6 fatty acids: LA (76 and 248 *μ*g/g DW) and AA (417 and 678 *μ*g/g DW), the values in parenthesis are for stipe and blade, respectively. The ratios between *n*-6 and *n*-3 fatty acids are ≤4.4/1 in all lipid fractions but especially low (≤1.3/1) in blade. The blades also presented the highest PUFA/SFA ratio. Regarding the potential of commercialization in respect of nutritional applications of *L. hyperborea*, blade is found to represent the most suitable material, due to higher levels of PUFAs and a low *n*-6/*n*-3 ratio.

## Figures and Tables

**Figure 1:**
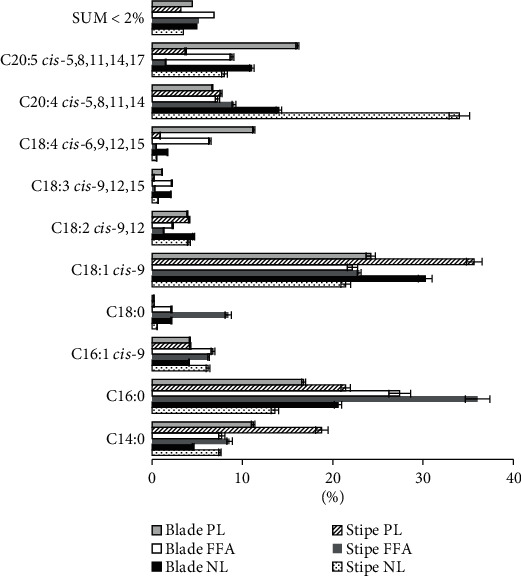
Fatty acid profile for fatty acids contributing more than 2% of the total fatty acid content, in at least one lipid fraction. *SUM* < 2% is the summarized contribution of the remaining fatty acids. (*n* = 4, two injection parallels for each of these four sample replicates, *error* *bars* = ±*SD*). NL: neutral lipid; FFA: free fatty acid; PL: polar lipid.

**Figure 2:**
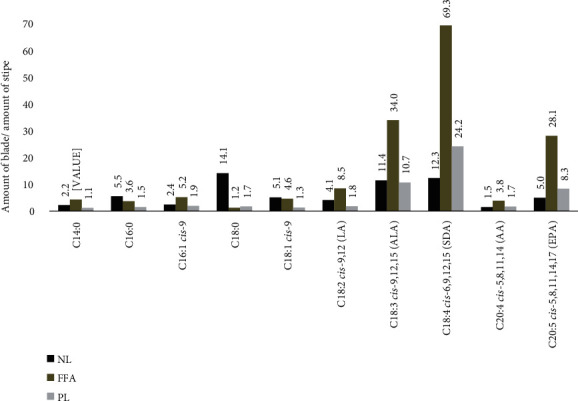
Ratio between average blade and stipe amounts in the predominating fatty acids. NL: neutral lipid; FFA: free fatty acid; PL: polar lipid.

**Table 1:** Fatty acid content in the lipid fractions (*mean* ± *SD*, *μ*g/g DW) of stipe and blade from *L. hyperborea* (*n* = 4, two injection parallels for each of these four sampling events).

Fatty acid		Stipe			Blade		
		NL	FFA	PL	NL	FFA	PL
C7 : 0 5 methyl^a^		0.61 ± 0.04	n.d.	n.d.	0.23 ± 0.01	n.d.	n.d.
C8 : 0		0.68 ± 0.05	0.34 ± 0.02	n.d.	2.48 ± 0.29	0.31 ± 0.01	n.d.
C9 : 0		n.d.	0.22 ± 0.03	n.d.	n.d.	0.34 ± 0.02	n.d.
C10 : 0		0.42 ± 0.03	n.d.	n.d.	2.76 ± 0.35	0.22 ± 0.03	n.d.
C12 : 0		0.34 ± 0.06	1.11 ± 0.15	0.06 ± 0.01	0.47 ± 0.02	1.93 ± 0.09	0.04 ± <0.00
C13 : 0		n.d.	0.14 ± 0.01	n.d.	0.12 ± 0.01	0.69 ± 0.02	0.04 ± <0.00
C14 : 0		74.33 ± 1.32	16.79 ± 0.60	152.06 ± 5.54	164.25 ± 3.41	72.21 ± 3.31	174.39 ± 3.13
C14 : 0 13 methyl		n.d.	0.27 ± 0.04	n.d.	n.d.	0.32 ± 0.01	n.d.
C14 : 1 *trans-*9		n.d.	0.15 ± 0.01	n.d.	n.d.	1.43 ± 0.09	n.d.
C14 : 1 *cis-*9		0.65 ± 0.03	0.10 ± 0.01	0.15 ± 0.02	1.78 ± 0.02	0.35 ± 0.01	0.21 ± 0.01
C15 : 0		0.74 ± 0.04	0.81 ± 0.04	1.49 ± 0.02	6.80 ± 0.14	5.20 ± 0.15	6.18 ± 0.08
C16:0		134.62 ± 4.19	70.62 ± 2.65	173.40 ± 4.33	741.81 ± 14.71	256.99 ± 11.23	261.56 ± 3.38
C16 : 1^b^		n.d.	n.d.	n.d.	0.63 ± 0.08	1.12 ± 0.03	0.40 ± 0.01
C16 : 1^b^		n.d.	0.45 ± 0.03	0.23 ± 0.03	1.83 ± 0.08	2.35 ± 0.08	0.66 ± 0.01
C16 : 1 *cis-*9		61.31 ± 2.09	12.26 ± 0.18	33.95 ± 0.64	146.88 ± 1.58	63.23 ± 1.94	65.17 ± 0.71
C17 : 0		0.28 ± 0.02	0.47 ± 0.06	0.38 ± 0.03	5.76 ± 0.21	1.58 ± 0.06	1.21 ± 0.03
C16 : 2 *cis* or *trans-*7,10^a^	(*n*-6)	0.50 ± 0.03	0.11 ± 0.02	0.30 ± 0.04	1.63 ± 0.05	1.46 ± 0.04	1.56 ± 0.03
C17 : 1 *cis-*9		0.29 ± 0.03	0.09 ± 0.04	0.23 ± 0.02	3.57 ± 0.21	1.82 ± 0.05	1.29 ± 0.04
C18 : 0		5.44 ± 0.26	16.54 ± 0.68	1.76 ± 0.06	76.79 ± 1.84	20.05 ± 0.72	3.08 ± 0.04
C18 : 1 *cis-*9		212.52 ± 5.32	44.93 ± 0.41	288.67 ± 7.13	1089.76 ± 27.19	207.93 ± 5.25	377.59 ± 8.00
C18 : 1 *cis-*11		3.06 ± 0.28	2.15 ± 0.04	3.35 ± 0.16	10.34 ± 0.19	6.28 ± 0.15	5.30 ± 0.10
C18 : 2 all-*cis-*9,12 (LA)^c^	(*n*-6)	40.26 ± 1.59	2.54 ± 0.08	33.18 ± 0.69	165.54 ± 3.78	21.48 ± 0.66	60.72 ± 0.82
C18 : 3 all-*cis-*6,9,12	(*n*-6)	2.89 ± 0.19	n.d.	2.45 ± 0.09	12.55 ± 0.16	2.82 ± 0.06	8.37 ± 0.13
C20 : 0		6.70 ± 0.54	1.42 ± 0.07	1.16 ± 0.07	49.81 ± 1.20	10.28 ± 0.28	3.66 ± 0.07
C18 : 3 all-*cis-*9,12,15 (ALA)^d^	(*n*-3)	6.54 ± 0.28	0.61 ± 0.03	1.61 ± 0.10	74.77 ± 1.23	20.57 ± 0.51	17.17 ± 0.46
C20 : 1 *cis-*9		n.d.	0.41 ± 0.08	2.53 ± 0.13	7.86 ± 0.40	4.34 ± 0.06	7.40 ± 0.14
C20 : 1 *cis-*11		n.d.	0.17 ± 0.04	0.29 ± 0.04	0.89 ± 0.05	0.41 ± 0.02	0.72 ± 0.02
C18 : 4 all-*cis-*6,9,12,15 (SDA)^e^	(*n*-3)	4.96 ± 0.23	0.86 ± 0.03	7.26 ± 0.16	61.22 ± 1.15	59.86 ± 1.33	175.42 ± 1.87
C20 : 2 all-*cis-*11,14	(*n*-6)	3.73 ± 0.35	0.27 ± 0.05	3.68 ± 0.14	10.27 ± 0.33	2.95 ± 0.12	6.46 ± 0.13
C20 : 3^b^		1.90 ± 0.16	0.12 ± 0.02	0.67 ± 0.05	1.88 ± 0.05	0.51 ± 0.03	0.81 ± 0.02
C20 : 3^b^		1.46 ± 0.13	0.07 ± 0.01	0.78 ± 0.04	4.43 ± 0.58	0.74 ± 0.04	2.03 ± 0.04
C20 : 3^b^		n.d.	0.04 ± 0.01	0.32 ± 0.02	1.19 ± 0.62	0.29 ± 0.02	0.59 ± 0.02
C20 : 3 all-*cis-*8,11,14	(*n*-6)	3.58 ± 0.34	0.16 ± 0.02	3.89 ± 0.15	17.95 ± 1.27	0.82 ± 0.02	3.34 ± 0.06
C22 :0		n.d.	0.07 ± 0.02	n.d.	1.02 ± 0.14	0.48 ± 0.03	n.d.
C20 : 4 all-*cis-*5,8,11,14 (AA)^f^	(*n*-6)	337.17 ± 11.22	17.75 ± 0.40	61.70 ± 1.11	505.89 ± 11.27	67.92 ± 2.21	104.14 ± 0.93
C20 : 4^b^		n.d.	n.d.	0.33 ± 0.03	2.74 ± 0.13	0.94 ± 0.04	2.39 ± 0.05
C20 : 4 all-*cis-*8,11,14,17	(*n*-3)	3.22 ± 0.25	n.d.	1.78 ± 0.10	13.23 ± 0.32	3.84 ± 0.18	7.53 ± 0.13
C20 : 5 all-*cis-*5,8,11,14,17 (EPA)^g^	(*n*-3)	79.44 ± 3.18	2.95 ± 0.07	30.04 ± 0.64	397.87 ± 9.25	82.87 ± 2.02	250.75 ± 2.66
C24 : 0		n.d.	0.36 ± 0.03	n.d.	0.75 ± 0.09	1.79 ± 0.08	n.d.
C22 : 5 all-*cis-*7,10,13,16,19	(*n*-3)	3.20 ± 0.29	0.19 ± 0.01	1.26 ± 0.08	16.33 ± 0.53	5.48 ± 0.12	9.12 ± 0.21
C22 : 6 all-*cis-*4,7,10,13,16,19 (DHA)^h^	(*n*-3)	n.d.	0.09 ± 0.02	0.27 ± 0.05	n.d.	1.84 ± 0.07	0.18 ± 0.02
C26 : 0		n.d.	0.35 ± 0.03	n.d.	n.d.	1.43 ± 0.09	n.d.
Total		990.87	195.98	809.24	3604.08	937.44	1559.49

^a^Fatty acids are identified by NIST library search only; ^b^unknown isomer of fatty acid, identified by NIST library search only; ^c^LA: linoleic acid; ^d^ALA: alpha linolenic acid; ^e^SDA: stearidonic acid; ^f^AA: arachidonic acid; ^g^EPA: eicosapentaenoic acid; ^h^DHA: docosahexaenoic acid; n.d.: not detected. Abbreviations: NL: neutral lipid; FFA: free fatty acid; PL: polar lipid.

**Table 2:** Sum of SFAs, MUFAs, and PUFAs, as well as *n*-6 and *n*-3 in *L. hyperborea* given in *μ*g/g DW, (*n* = 4, two injection parallels for each sample replicate).

	Stipe			Blade		
	NL	FFA	PL	NL	FFA	PL
*Σ* SFA	224.16 ± 4.19	109.51 ± 2.65	330.32 ± 5.54	1053.05 ± 14.71	373.81 ± 11.23	450.16 ± 3.38
*Σ* MUFA	277.83 ± 5.32	60.70 ± 0.41	329.40 ± 7.13	1263.54 ± 27.19	289.24 ± 5.25	458.74 ± 8.00
*Σ* PUFA	488.88 ± 11.22	25.77 ± 0.40	149.52 ± 1.11	1287.49 ± 11.27	274.39 ± 2.21	650.58 ± 2.66

Total	990.87	195.98	809.24	3604.08	937.44	1559.49

*Σn*-3	97.37 ± 3.18	4.71 ± 0.07	42.22 ± 0.64	563.42 ± 9.25	174.46 ± 2.02	460.17 ± 2.66
*Σn*-6	388.14 ± 11.22	20.84 ± 0.40	105.20 ± 1.11	713.83 ± 11.27	97.44 ± 2.21	184.59 ± 0.93
*n*-6/*n*-3	3.99	4.43	2.49	1.27	0.56	0.40
PUFA/SFA	2.18	0.24	0.45	1.22	0.73	1.45

The standard deviations are the highest standard deviation among the summarized values. NL: neutral lipid; FFA: free fatty acid; PL: polar lipid; SFA: saturated fatty acid; MUFA: monounsaturated fatty acid; PUFA: polyunsaturated fatty acid.
